# A comparative study on rumen ecology of water buffalo and cattle calves under similar feeding regime

**DOI:** 10.1002/vms3.302

**Published:** 2020-07-13

**Authors:** Qiyan Wang, Xiaomei Gao, Yunyan Yang, Caixia Zou, Yingbai Yang, Bo Lin

**Affiliations:** ^1^ College of Animal Science Guangxi University Nanning China

**Keywords:** comparative study, fermentation, growth performance; water buffalo, rumen microbiome

## Abstract

In all, 12 male water buffalo (*Bubalus* *bubalis*) calves and Holstein (*Bos* *taurus*) calves of similar age (10 ± 5 days) were selected to explore the mechanism underlying the differences in growth performance and feed conversion ratio between the two species. The experiment contains 33 days of sucking period and 40 days of post‐weaning period. Both calves were fed the same amounts of milk in sucking period, and starter and oat grass were supplied ad libitum both before and after the weaning period. Feed intake, growth performance, ruminal fermentation parameters and the ruminal microbial community were measured the during experiment period. Results showed no differences in growth performance and feed intake between the two species in sucking period; however, the feed/gain ratio (F/G) of the water buffalo was higher than that of Holstein calve (*p* > 0.05). After weaning, the intake of starter by the Holstein calf was higher while intake of grass by the water buffalo was higher resulting in higher growth performance of and a lower F/G ratio for Holstein (*p* < 0.05). The rumen of Holstein calf showed higher levels of propionate, lower levels of acetate and branched‐chain fatty acids than that of water buffalo during both periods (*p* < 0.05). The rumen of water buffalo showed a higher number of observed bacterial species and Shannon diversity as compared with that of Holstein calf. The members belonging to the bacterial phylum *Bacteroides* and genus *Prevotella* in the rumen of Holstein calf were higher (*p* < 0.05), while Firmicutes and fibrolytic bacteria *Ruminobacter* and *Ruminococcus* were lower (*p* < 0.05) than that of water buffalo. In conclusion, the water buffalo calves demonstrated clearly of having significant population of bacterial community and better fibre digestion than those of cattle calves.

## Graphical abstract

Differences in growth performance and rumen microbial community between water buffalo and Holstein calves under similar feeding conditions: a comparative study.

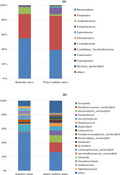

## 1 INTRODUCTION

The water buffalo and the Holstein cows are very important livestock species bred primarily for milk supply. Water buffalo are mainly grown in Asian countries, such as India, Pakistan and China (Coffey, Horan, Evans, & Berry, 2019). Currently, water buffalo are mainly used for milk and beef, which explains the increased interest in their growth and lactating performance in recent years. Studies have shown that water buffalo digest roughage, especially feed containing high acid detergent fibre (ADF), higher than the cattle, via mechanisms unique to the ruminal microflora (Chanthakhoun, Wanapat, Kongmun, & Cherdthong, 2012), such as higher population of cellulolytic bacteria, fungal zoospores and lower protozoa in the buffalo rumen compared with the cattle rumen. Previous study also demonstrated differences in the rumen microbial population and community of adult water buffalo and Jersey cows, higher populations of bacteria, protozoa and fungi, which explain the higher efficiency of fibre digestion by a buffalo (Iqbal et al., 2018); however, the mechanisms underlying the differences were unclear.

Studies showed the presence of ruminal microbes in lambs before ingestion of solid feed (2–4 days), resulting in a composition similar to that of adult animals around 10 days after birth (Morvan et al., 1994; Stewart, Fonty, & Gouet, 1988). Therefore, the rumen microbiome established early in a ruminant's life determines the ruminal bacterial community and nutrient digestibility in the adult period ( Leahy et al., 2013; Yáñez‐Ruiz & Martín‐García, 2016). However, no studies investigated the differences between water buffalo and cattle at the calf stage. Holstein is a typical cattle breed genetically selected for high feed intake and production; therefore, it can be regarded as an animal with tolerance for high concentrate feed compared with water buffalo. Prihantoro, Toharmat, Evvyernie, and Suryani (2012) reported that inoculation of fibre‐digesting bacteria from a water buffalo calf into the rumen of 2‐week‐old Holstein calf increased its feed intake, protein digestibility and the number of rumen bacteria, indicating the differences in ruminal microbial composition between water buffalo and Holstein calf probably during the sucking period. However, the effect of differences between bacterial communities on the feed intake and growth performance of buffalo, and the variation in ruminal bacterial community with animal growth remains unknown.

In this study, we compared the differences in feed intake, growth performance and ruminal fermentation parameters between water buffalo and Holstein calves exposed to a similar diet both before and after weaning. The differences in the composition of ruminal bacterial community in the two animal species were measured to explore the effects on their feed intake, growth and immunity.

## 2 MATERIALS AND METHODS

### 2.1 Ethical statement

The animals used in this study were housed at the experimental animal farm of (blind for review). All the experimental protocols regarding animal handling and treatment were approved by the (blind for review).

### 2.2 Animals, diets and management

In all, 12 male water buffalo calves (*Bubalus bubalis*), a hybrid between the Murrah and the local Chinese buffalo, and 12 male Holstein (*Bos taurus*) calves were selected for this study. All animals were at similar age (10 ± 5 days) and body weight, the water buffalo calf was 50 ± 2.1 kg, and Holstein calf was 50 ± 1.2 kg. Each group of animals was separated into three subgroups, each containing four animals. All the animals were fed in clean and comfortable animal house in Farm of (blind for review), 5 m^2^ area. They were fed on same amount of whole milk powder as milk replacer (fat content is 20% and protein is 23%, DM basis), the feeding procedure was as follows: 6 L of milk which diluted by mixing whole milk powder with water at 1:7 ratio was fed for 2 weeks, 8 L of milk was fed for 2 weeks, 4 L for 1 week and 2 L for about 1 week, finally weaning on day 43. Besides, the oat grass and starter were accessed by all calves ad libtium during the sucking period. During the whole experiment period, the starter and the oat grass were given separate to ensure adequate intake. Furthermore, clean drinking water was provided ad libitum. After weaned, both the animal groups were fed with starter and dry oat grass ad libitum for another 40 days feeding experiment. The composition of starter diet was as follows (based on dry matter): maize 54.4%, wheat bran 10%, soybean meal 22%, expanded soybean 5%, whey powder 2.5%, sugar 2.5%, calcium hydrogen phosphate 2.5% and premix 0.6%. Each kg of the premix contained 19 g MgSO_4_·H_2_O; 25 g FeSO_4_·7H_2_O; 8 g CuSO_4_·5H_2_O; 30 g MnSO_4_·H_2_O; 15 g ZnSO_4_·H_2_O; 100 mg Na_2_SeO_3_; 400 mg KI; 300 mg CoCl_2_·6H_2_O; Vitamin A 1,250,000 IU; Vitamin D3 30,000 IU and Vitamin E 1,800 IU. The chemical composition of oat grass was as follows: dry matter 88.2%, neutral detergent fibre 55.7%, ADF 39.2%, crude protein 4.5% and water‐soluble carbohydrate 20.5%. The feed refusals, including oat grass and starter, was cleaned every 2 days to ensure a fresh diet, and the feed intake was recorded.

### 2.3 Growth performance, diets and management

During the experiment, we totally weight the calve three times, the first time is at the beginning of the experiment, second time is weaning day and the third time is at the end of experiment, and each time was weighed in the morning before feeding, and the parameters including body height, body length, body oblique length, chest circumference and cannon circumference were measured. The ruminal fluid (50 ml) was drawn using a gastric tube from seven animals in each group, filtered with a four‐layered cheese‐cloth and frozen in −80℃ immediately before been used to measure the ruminal volatile fatty acid (VFA) composition. Both the animal groups were fed with the starter diet and dry oat grass ad libitum for another 40 days after weaning. At the end of the experiment on day 40, the body weights of all the animals were measured. All the parameters were measured before feeding in the morning, and the ruminal fluid (50 ml) of seven animals in each group was drawn via gastric tube. The ruminal fluid was separated into two aliquots, with one aliquot used to measure ruminal fermentation parameters, and another aliquot for the measurement of ruminal bacterial composition via high‐throughput sequencing.

### 2.4 Rumen VFA analysis and high‐throughput sequencing

The concentration of ruminal VFA was determined using a gas chromatograph (GC‐2010; Shimadzu), equipped with a flame ionization detector and a capillary column (HP‐INNOWAX, 1909N‐133; Agilent Technologies) as described by Zhang et al. (2008). Rumen microbial DNA was extracted from 1.5 ml of the preserved sample, for high‐throughput sequencing. The DNA extraction was performed as described by Rius et al. (2012). The bacterial 16S rRNA genes were amplified using the specific primer V4–V5: 515F(GTGCCAGCMGCCGCGG)‐907R(CCGTCAATTCMTTTRAGT) with the barcode. All PCR reactions were carried out in 30 µl reaction volumes mixed with 15 µl of Phusion^®^High‐Fidelity PCR Master Mix (New England Biolabs), 0.2 µM of forward and reverse primers, and about 10 ng of template DNA. All the PCR products were mixed in equidensity ratios. The PCR products in the mixture were purified with a GeneJET Gel Extraction Kit (Thermo Scientific). Sequencing libraries were generated using NEB Next^®^Ultra™DNA Library Prep Kit for Illumina (NEB) and the library was sequenced on an Illumina MiSeq PE 300 platform.

Sequence analysis was performed using the UPARSE software (Edgar, 2013) package with the UPARSE‐OTUout and UPARSE‐OTUref algorithms. In‐house Perl scripts were used to analyse alpha (within samples) and beta (among samples) diversity. Sequences with ≥97% similarity were assigned to the same operational taxonomic units (OTUs). We selected representative sequences of each OTU and used the RDP classifier (Wang, Garrity, Tiedje, & Cole, 2007) to annotate the taxonomic information for each representative sequence. Bacterial data were summarized at the phylum and the genus levels. To compute alpha diversity, we rarified the OTU table and calculated the three metrics for each observed species: Chao1, Shannon index and Simpson index. Respective bar charts for bacteria both at phylum and genus levels were drawn. Venn diagrams were also designed to present the OUT analysis of the bacterial species.

### 2.5 Statistical analysis

All the preliminary data including feed intake, growth performance, body measurements, rumen fermentation parameters, bacterial diversity and bacterial relative abundances at phylum and genus levels were sorted using Microsoft Excel 2013, and statistically analysed with SAS 8.02 software using a one‐way factorial design of ANOVA procedure. Differences among means were tested using Duncan's multiple range tests. Significant differences were achieved when *p* < 0.05.

## 3 RESULTS

### 3.1 Growth performance, feed intake and body measurement

No differences existed in growth and feed intake parameters between Holstein and water buffalo calves during sucking period; however, the average daily gain and dry matter intake of oat grass daily by the Holstein calves tended to be higher (Table 1, *p* > 0.05), and the feed/gain ratio (F/G) was lower than that of the water buffalo before weaned (Table 1, *p* < 0.05). After weaned, the dry matter intake of starter by the Holstein calves was higher (*p* < 0.05); however, the dry matter intake of oat grass the Holstein calves was lower than that of the water buffalo calves (Table 1, *p* < 0.05). Generally, a higher average daily gain and lower F/G ratio for Holstein calves was observed as compared with the buffalo both during the period of after weaned and whole experiment (*p* < 0.05). The parameters of body height, length, oblique length and chest circumference of the Holstein calves were higher than that of water buffalo calves (Table 2, *p* < 0.05), whereas the cannon circumference was lower than that of water buffalo at every time point, including start, weaning and end of the day (Table 2, *p* < 0.05).

**TABLE 1:** The growth performance of Holstein calves and water buffalo calves

Items	Holstein calves	Water buffalo calves	*SEM*	*p*
Before weaned				
Initial body weight (kg)	50.0	52.6	1.782	0.666
Final body weight (kg)	69.1	67.1	3.717	0.576
Average daily gain	0.43	0.34	0.062	0.288
Dry matter intake of starter (kg/day)	0.56	0.56	0.112	0.696
Dry matter intake of oats (kg/day)	0.11	0.09	0.255	0.511
Dry matter intake of milk replacer (kg/day)	0.58	0.58	0.000	1.000
Feed/gain	2.90^a,b^	3.61^a,b^	0.150	0.013
After weaned				
Initial body weight (kg)	69.1	67.1	3.717	0.576
Final body weight (kg)	110^a,b^	91.1^a,b^	5.805	0.005
Average daily gain	0.98^a,b^	0.57^a,b^	0.079	<0.001
Dry matter intake of starter (kg/day)	2.12^a,b^	1.63^a,b^	0.085	0.008
Dry matter intake of oats (kg/day)	0.23^a,b^	0.37^a,b^	0.052	0.029
Feed/gain	2.48^a,b^	3.50^a,b^	0.190	<0.001
Whole period				
Average daily gain	0.73^a,b^	0.46^a,b^	0.062	<0.001
Dry matter intake of starter (kg/day)	1.31^a,b^	1.06^a,b^	0.081	0.004
Dry matter intake of oats (kg/day)	0.16^a,b^	0.23^a,b^	0.042	0.029
Feed/gain	2.01^a,b^	2.80^a,b^	0.025	<0.001

^a,b^ Within a row, means with different superscripts differ at *p* < 0.05.

**TABLE 2:** Body measurement parameters of Holstein calves and water buffalo calves

Items	Holstein calves	Water buffalo calves	*SEM*	*p*
Starting day				
Body height (cm)	81.2^a,b^	75.0^a,b^	1.949	0.008
Body length (cm)	66.3	63.0	1.463	0.077
Body oblique length (cm)	77.5^a,b^	71.1^a,b^	1.513	<0.001
Chest circumference (cm)	88.0^a,b^	85.3^a,b^	1.121	0.036
Cannon circumference (cm)	11.5^a,b^	13.4^a,b^	0.193	<0.001
Weaning day				
Body height (cm)	87.7^a,b^	82.1^a,b^	1.433	0.005
Body length (cm)	78.0^a,b^	71.6^a,b^	1.445	<0.001
Body oblique length (cm)	84.0^a,b^	78.1^a,b^	1.649	0.004
Chest circumference (cm)	97.5	93.5	2.355	0.131
Cannon circumference (cm)	12.1b	13.1^a,b^	0.306	0.012
Finishing day				
Body height (cm)	96.3^a,b^	85.5^a,b^	1.529	<0.001
Body length (cm)	93.7^a,b^	80.2^a,b^	1.922	<0.001
Body oblique length (cm)	100^a,b^	86.2^a,b^	2.458	<0.001
Chest circumference (cm)	113^a,b^	106^a,b^	2.184	0.013
Cannon circumference (cm)	13.7^a,b^	15.3^a,b^	0.333	<0.001

^a,b^ Within a row, means with different superscripts differ at *p* < 0.05.

### 3.2 Rumen fermentation parameters and bacterial community analysis

**FIGURE 1:**
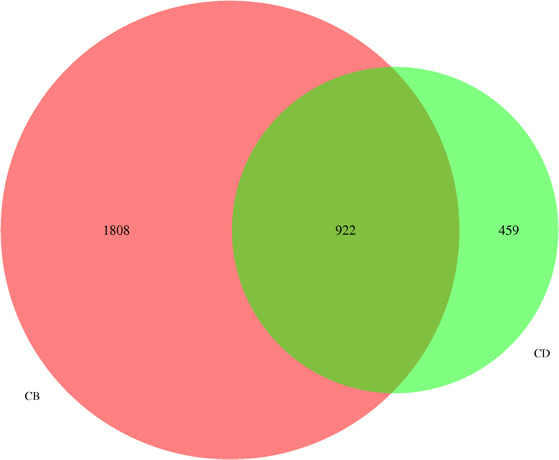
Venn diagram of OTU distribution of ruminal bacterial community in Holstein calves and water buffalo calves

**FIGURE 2:**
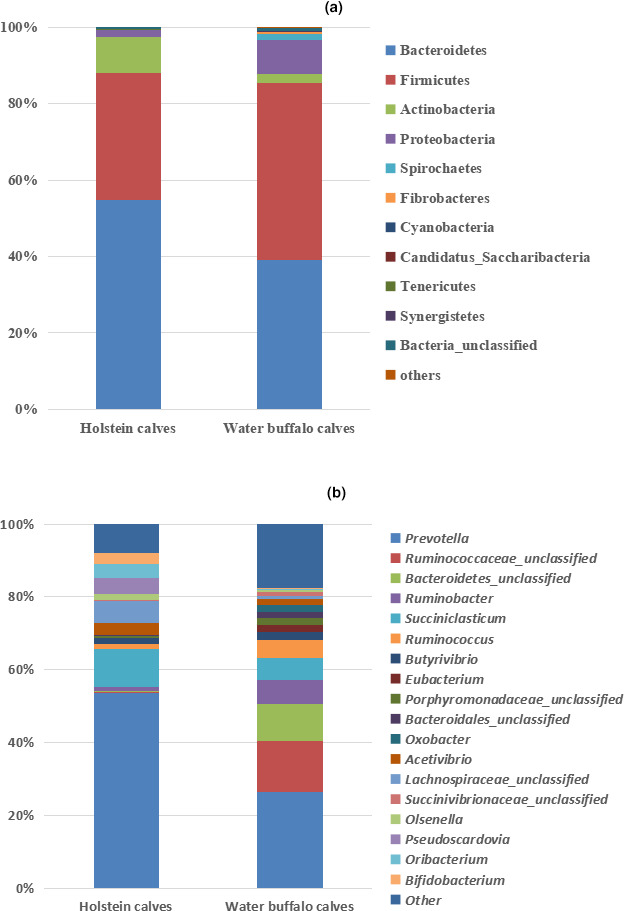
The relative abundance of the most abundant bacterial phyla (a) and genus (b) in rumen of Holstein calves and water buffalo calves

There is a significant difference in the bacterial abundance of rumen bacterial community in buffalo and cattle (Figure 1). On the weaning day, the level of ruminal fermentation parameters including ruminal propionate, valerate of Holstein calves were higher (*p* < 0.05), while parameters including, acetate, isobutyrate, isovalerate and acetate‐to‐propionate ratio were lower than that of water buffalo calves (Table 3, *p* < 0.05). On the final day, the VFA profile of both animal species was similar to the profile on the weaning day (Table 3), which appeared as higher level of acetate, isobutyrate, isovalerate and acetate‐to‐propionate ratio in rumen of water buffalo (*p* < 0.05). However, the butyrate and total VFA concentration were similar in both animal species (*p* > 0.05). As for ruminal bacterial community analysis, the indices of ruminal bacterial diversity including the observed species, Shannon and Chao1 induces of water buffalo calves were higher than those of Holstein calves (Table 4, *p* < 0.05). As can be seen from Figure 1, the ruminal bacteria OTU number of water buffalo were much higher than that of Holstein calve, and there were 922 OTU share by both species. At the phylum level, the abundance of Bacteroidetes and Actinobacteria in rumen of Holstein calves was higher (*p* < 0.05), while the Firmicutes was lower than that of water buffalo calves (Table 4; Figure 2, *p* < 0.05). Abundance of Bacterial genus including Ruminobacter, Ruminococcus and Ruminococcaceae_unclassified was lower (*p* < 0.05), while abundance of Bacterial genus including Prevotella, Lachnospiraceae_unclassified, Pseudoscardovia, Oribacterium, Bifidobacterium, Acetivibrio and Olsenella was higher in rumen Holstein calves as compared to that of Holstein calves (Table 4; Figure 2, *p* < 0.05).

**TABLE 3:** Rumen volatile fatty acids of Holstein calves and water buffalo calves

Items	Holstein calves	Water buffalo calves	*SEM*	*p*
Weaning day				
Acetate (mmol/L)	23.2^a,b^	32.9^a,b^	3.076	0.023
Propionate (mmol/L)	18.5^a,b^	11.5^a,b^	2.469	0.017
Butyrate (mmol/L)	3.60	3.65	0.900	0.950
Valerate (mmol/L)	1.57^a,b^	0.85^a,b^	0.230	0.010
Isobutyrate (mmol/L)	0.46^a,b^	0.76^a,b^	0.115	0.025
Isovalerate (mmol/L)	0.64^a,b^	1.31^a,b^	0.160	0.002
Acetate/propionate	1.28^a,b^	2.87^a,b^	0.308	0.002
Total VFA (mmol/L)	48.0	51.0	2.900	0.710
Finishing day				
Acetate (mmol/L)	26.9^a,b^	34.9^a,b^	3.870	0.038
Propionate (mmol/L)	17.6^a,b^	10.5^a,b^	2.160	0.006
Butyrate (mmol/L)	3.94	3.27	0.883	0.457
Valerate (mmol/L)	1.58^a,b^	0.76^a,b^	0.258	0.010
Isobutyrate (mmol/L)	0.57^a,b^	0.85^a,b^	0.125	0.038
Isovalerate (mmol/L)	0.80^a,b^	1.73^a,b^	0.259	0.003
Acetate/propionate	1.57^a,b^	3.66^a,b^	0.459	0.002
Total VFA (mmol/L)	53.0	52.8	2.920	0.942

Abbreviation: VAF, volatile fatty acid.

^a,b^ Within a row, means with different superscripts differ at *p* < 0.05.

**TABLE 4:** Alpha diversity, relative abundance of rumen bacteria at phylum and genera level of Holstein calves and water buffalo calves

Items	Holstein calves	Water buffalo calves	*SEM*	*p*
Bacterial alpha diversity index				
Observed species	496^a,b^	794^a,b^	43.30	<0.001
Shannon	4.53^a,b^	6.17^a,b^	0.144	<0.001
Simpson	0.85^a,b^	0.93^a,b^	0.009	0.001
Chao1	759.0	1,078	29.57	0.156
Abundance at phylum level (%)				
Bacteroidetes	54.9^a,b^	39.0^a,b^	2.295	<0.001
Firmicutes	33.1^a,b^	46.3^a,b^	0.468	0.002
Actinobacteria	9.41^a,b^	2.45^a,b^	1.287	0.023
Proteobacteria	1.94	9.00	0.324	0.338
Spirochaetes	0.01	1.62	1.228	0.528
Fibrobacteres	0.01	0.29	0.143	0.545
Cyanobacteria	0.03	0.05	0.010	0.010
Abundance at genus level (%)				
*Prevotella*	53.6^a,b^	26.5^a,b^	3.662	<0.001
*Succiniclasticum*	10.3	6.17	0.340	0.460
Lachnospiraceae_unclassified	6.02^a,b^	1.01^a,b^	3.006	0.008
*Pseudoscardovia*	4.23^a,b^	0.01^a,b^	2.034	0.034
*Oribacterium*	3.78^a,b^	0.34^a,b^	1.441	<0.001
*Bifidobacterium*	3.25^a,b^	0.03^a,b^	1.067	<0.001
*Acetivibrio*	3.21^a,b^	1.63^a,b^	0.809	<0.001
*Olsenella*	1.88^a,b^	0.68^a,b^	0.803	<0.001
*Butyrivibrio*	1.69	2.09	0.390	0.334
*Ruminobacter*	1.35^a,b^	6.64^a,b^	0.387	0.005
*Ruminococcus*	1.29^a,b^	4.94^a,b^	1.182	0.001
Ruminococcaceae_unclassified	0.22^a,b^	14.02^a,b^	3.716	0.006

^a,b^ Within a row, means with different superscripts differ at *p* < 0.05.

## 4 DISCUSSION

Several studies analysed the differences in growth and digestion between animal species or breeds, to explore the mechanism of nutrient digestion, for possible genetic improvement, while the difference in digestion between animal species was easy to investigate in the juvenile period. In this study, the absence of differences in growth performance and feed intake between water buffalo and Holstein before weaning under same environment, indicating that both animals exhibit similar utilization efficiency for milk replacer, which is the main nutritional source of both animals. The Holstein calf tended to show a higher intake of oat grass and average daily gain than the water buffalo calf during the sucking period probably due to long‐term genetic selection resulting in feed efficiency and growth performance in Holstein (Berry et al., 2014), unlike water buffalo. However, the ruminal fermentation parameters on the weaning day differed between the two animal species, mainly reflected by ruminal acetate/propionate ratio, and the higher levels of branched chain fatty acids in the water buffalo calf than in the Holstein calf. Lower acetate/propionate ratio in rumen of Holstein calves indicating that the Holstein calves exhibited propionate producing fermentation, which can utilizing ruminal hydrogen produced by ruminal carbohydrate degradation, resulting in higher feed conversion rates (Wang, Sun, Janssen, Tang, & Tan, 2014). The higher propionate in rumen of Holstein calf is in agreement with the *Prevotella* spp. which is a group of bacteria responsible for propionate and succinate (propionate precursor) production was much higher in rumen of Holstein calf (Wang et al., 2016). The higher acetate/propionate ratio in rumen of buffalo calves was probably a result of the increase oat grass intake in Buffalo, indicating the ruminal microbiota of buffalo are adapted to a roughage environment, and therefore they increase the grass consumption. The branched‐chain fatty acids (BCFA) can be utilized by ruminal microbes to synthesize the cell membrane (Felix, Cook, & Huber, 1980); therefore, higher BCFA can promote the growth of ruminal fibrolytic bacteria and increasing ruminal fibre degradation (Yang, 2002). We can predicted the high intake of roughage and digestibility of water buffalo was probably related to its high ruminal branch‐chain fatty acid production promoted its growth of ruminal bacteria, which is in consistent with a study showed population of ruminal bacteria was higher than cattle (Iqbal et al., 2018).

Differences in feed intake and growth performance between the two animal species were observed after the weaning period. The Holstein calf showed a substantially higher intake of starter diet which contains much higher protein and metabolism energy than oat grass, whereas the water buffalo calf consumed significantly higher oat grass, which resulted in higher growth performance of Holstein calf than the water buffalo. Furthermore, almost all the body size parameters of Holstein calf were higher than that of water buffalo calf except for the cannon circumference, further suggesting that genetic selection strongly enhanced the growth performance of Holstein (Koenen & Groen, 1998). Due to the differences in feed intake, higher propionate concentration in the rumen of Holstein calf was attributed to higher concentrate intake, while the higher acetate in the rumen of water buffalo calf was attributed to the higher intake of oat grass, which is in consistent with other studies showed Ruminants fed a grain diet produce more propionate as a proportion than those fed a forage diet (Beauchemin & McGinn, 2005; Christophersen, Wright, & Vercoe, 2008). Acetate is a synthetic precursor of fatty acid in the body of ruminants, with the higher acetate in the rumen of water buffalo calf suggesting higher levels of body fat synthetize as compared with the Holstein calf, besides, acetate production in rumen implies higher hydrogen and methane production which lead to lower feed energy conversation efficiency for ruminants (Janssen, 2010). Therefore, the F/G ratio of Holstein calf was higher than that of water buffalo calf due to both ruminal propionate fermentation have higher feed energy utilization efficiency and lower body fat synthesis in body. The factors underlying the high concentration of BCFA in the rumen of water buffalo are unknown and are probably related to the unique microbial composition of the buffalo rumen.

Studies suggest that the ruminal bacterial community was individual‐ or host‐specific (Weimer, 2015), which explains the differences between the water buffalo and Holstein calves, and is supported by the results of Chanthakhoun et al. (2012) and a previous study (Iqbal et al., 2018). In this study, the observed bacterial species and the Shannon diversities of the water buffalo calf were higher than that of the Holstein calf, indicating a higher diversity of the ruminal bacteria of water buffalo. Furthermore, the ruminal bacteria of water buffalo calf were dominated by members belonging to the phylum Firmicutes, while the Holstein calf was dominated by phylum Bacteroidetes. The Firmicutes/Bacteroidetes ratio in the animal digestive tract plays an important role in nutrients digestibility of digest tract and the development of obesity, and a high ratio facilitated nutrient digestion and body fat deposition (Bäckhed, Ley, Sonnenburg, Peterson, & Gordon, 2005; Bajzer & Seeley, 2006). The water buffalo calf showed a higher ruminal Firmicutes/Bacteroidetes ratio, further suggesting that the fermentation ability of ruminal microbes in buffalo rumen was probably higher than that of the Holstein calf, which also can be supported by the high bacterial diversity in rumen of buffalo, because high bacterial diversity is benefit for fibre degradation (Belanche et al., 2012).

At the genus level, the abundance of fibrolytic bacteria, such as *Ruminobacter*, *Ruminococcus* and Ruminococcaceae_unclass in the rumen of water buffalo calf was significantly higher, indicating that buffalo was more efficient in digesting dietary fibre, which was consistent with the study of Wanapat (2000). The abundance of *Prevotella* was much lower in the rumen of water buffalo calf than that of the Holstein calf consistent with a study involving compare between water buffalo and Jersey cow (Iqbal et al., 2018). *Prevotella* is a group of multifunctional bacteria, mainly participating in ruminal saccharolytic and proteolytic processes (Downes, Sutcliffe, Booth, & Wade, 2007; Jami, Israel, Kotser, & Mizrahi, 2013). Therefore, the high abundance of *Prevotella* in the Holstein rumen was required to degrade the dietary nutrients. In general, water buffalo calf tended to consume oat grass predominantly, leading to a higher bacterial diversity in the bacterial lumen and an abundance of fibrolytic bacteria, leading to the production of higher levels of ruminal acetate. By contrast, the Holstein calves tended to consume more on the starter, and its rumen enriched in saccharolytic and proteolytic bacteria generated higher levels of propionate, which was consistent with the dietary effect on the composition of rumen microbial community (Stevenson & Weimer, 2007). However, it is uncertain whether the oat grass preference of water buffalo was determined by its ruminal microbial community in this study, because animal feeding is attributed to long‐term evolution of animals under specific environments (Olff, Ritchie, & Prins, 2002; Wang, Wang, He, Liu, & Hodgkinson, 2001). The long‐term survival of subtropical areas rich in plant species is probably determined by the grass intake preferences of water buffalo.

## 5 CONCLUSION

This study suggests that the Holstein and water buffaloes carry different ruminal microflora and exhibit varying nutrient metabolism during the sucking period, despite similar growth performance. The rumen of water buffalo is rich in Firmicutes and fibrolytic bacteria, which accounted for the increased intake of oat grass and resulted in acetate production, while the ruminal of energy transition associated with acetate fermentation and higher fat synthesis after acetate absorption into body resulted in a lower growth performance and a higher F/G ratio in water buffalo compared with the Holstein calves.

## CONFLICT OF INTEREST

The authors declare the absence of any conflict of interest.

## AUTHOR CONTRIBUTION

**Qiyan Wang:** Formal analysis; Investigation; Writing‐original draft. **Xiaomei Gao:** Investigation; Methodology; Validation. **Yunyan Yang:** Data curation; Investigation; Writing‐review & editing. **Caixia Zou:** Conceptualization; Project administration; Software. **Yingbai Yang:** Conceptualization; Data curation; Funding acquisition; Resources; Software. **Bo Lin:** Conceptualization; Formal analysis; Funding acquisition; Methodology; Resources; Supervision; Visualization; Writing‐review & editing.

### PEER REVIEW

The peer review history for this article is available at https://publons.com/publon/10.1002/vms3.302.
